# Comparative Analysis of Quality Attributes and Flavor Profiles of Broccoli (*Brassica oleracea* var. *italica*) Stalk and Floret Juices Fermented by *Limosilactobacillus reuteri*

**DOI:** 10.3390/foods15091519

**Published:** 2026-04-27

**Authors:** Yingzhuo Zhou, Yuqing Sun, Daotong Li, Chen Ma, Fang Chen

**Affiliations:** 1 Key Laboratory of Fruits and Vegetables Processing, National Engineering Research Center for Fruit and Vegetable Processing, College of Food Science and Nutritional Engineering, Ministry of Agriculture, China Agricultural University, No.17, Qinghua East Road, Haidian District, Beijing 100083, China; zhouyingzhuo123@126.com (Y.Z.); sunyuqing98@126.com (Y.S.); lidaotong@cau.edu.cn (D.L.); 2 Engineering Research Centre for Fruits and Vegetables Processing, Ministry of Education, China Agricultural University, Beijing 100083, China

**Keywords:** broccoli juice, lactic acid bacteria, fermentation, nutrient composition, flavor

## Abstract

This study compared the physicochemical properties, nutritional composition, and flavor characteristics of broccoli stalk and floret juices fermented with *Limosilactobacillus reuteri* 18 (Lr18) to enhance the valorization of broccoli processing by-products. Four sample groups were analyzed: non-fermented stalks, fermented stalks, non-fermented florets, and fermented florets. After 48 h of fermentation, total viable counts and total phenolic content were slightly higher in florets than in stalks. Total titratable acids, total sugars, total soluble solids (TSS), total flavonoids, and vitamin C were initially higher in florets but decreased after fermentation in both groups. Organic acid analysis revealed that fermentation increased citric acid, reduced oxalic acid, and promoted the conversion of malic acid to lactic acid. Stalks contained higher levels of lactic and malic acids but lower citric acid than florets. Tryptophan content was higher in florets and was partially converted to indole derivatives after fermentation. Volatile compound analysis and sensory evaluation indicated that fermentation reduced fruity notes in florets while increasing acidic and sulfurous notes. In contrast, fermentation enhanced fruity and rounded notes in stalks while reducing pungency. These findings provide a scientific basis for developing fermented vegetable products with improved functional and sensory properties, particularly using broccoli stalks as a valuable by-product.

## 1. Introduction

Globally, approximately 1.3 billion tons of food are wasted annually along the food supply chain, from the agricultural to final consumption stages [1]. Fruits and vegetables represent one of the leading sources of food waste produced worldwide [2]. Broccoli (*Brassica oleracea* var. *italica*) is a nutrient-dense vegetable, yet approximately 30–40% of the plant biomass is discarded during processing, including stalks, non-commercial florets, and smaller amounts of leaves [3]. To date, effective utilization strategies for these by-products remain limited, leading to significant economic losses and environmental pollution [4,5,6]. This is particularly regrettable given that these by-products are rich in glucosinolates, flavonoids, vitamins, and phenolic acids [4], which have been associated with reducing the risk of coronary heart disease, preventing several types of cancer, improving glucose and lipid metabolism, and modulating immune and inflammatory responses [5,6]. Therefore, exploring how to utilize broccoli by-products to increase their value is of great significance to both the food industry and environmental sustainability [7,8,9].

Fermentation is a traditional method of processing vegetables and fruit. During fermentation, various substances undergo biotransformation by microorganisms such as lactic acid bacteria (LAB), yeast, and filamentous fungi [10]. LAB fermentation can enhance active components through microbial metabolism, including increased organic acids, free phenolic compounds, altered carotenoids, and boosted vitamin levels [11,12,13]. This process also produces exopolysaccharides, bacteriocins, and γ-aminobutyric acid [14]. In cabbage fermentation, thioglucoside degradation yields sulfur-containing bioactive compounds like ascorbigen and isothiocyanates [13]. Additionally, fermentation restructures flavor profiles by increasing the diversity and relative abundance of volatile compounds, mainly esters, alcohols, acids, aldehydes, and ketones [15,16]. These characteristics make fermentation a promising strategy for valorizing vegetable by-products, including broccoli stalks. Although challenges such as limited strain diversity, product homogenization, and low probiotic survival rates remain [17,18], LAB fermentation is a viable and effective approach for converting by-products into value-added products. Most studies have focused on the flavor characteristics of whole broccoli during fermentation [19]. There is very little research that systematically compares the flavor of broccoli roots and stems before and after fermentation.

In this study, a strain of *Limosilactobacillus reuteri* (Lr18) isolated from a traditional plant-based fermented beverage was used to ferment broccoli stalk and floret juices. The effects of fermentation on physicochemical properties, nutritional composition, and flavor characteristics were evaluated, with a specific focus on comparing the two tissues. Changes in viable counts, organic acids, phenolic compounds, flavonoids, vitamin C, tryptophan metabolites, and volatile organic compounds were systematically analyzed before and after fermentation. The results are intended to clarify the differential responses of broccoli stalks and florets to LAB fermentation and to provide a scientific basis for utilizing broccoli by-products in the development of fermented vegetable products.

## 2. Materials and Methods

### 2.1. Materials

The strain Lr 18 was obtained from the National Engineering Research Center for Fruit and Vegetable Processing (Beijing, China), originally isolated from Douzhi (a traditional fermented beverage from Beijing, China, made from ground mung beans, which has a unique sour flavor and is rich in probiotics). The 16S rRNA gene sequence was deposited in NCBI GenBank (accession PX470202, submission SUB15725043). Fresh broccoli was purchased from a local agricultural market in Beijing.

### 2.2. Fermentation of Broccoli Juice

Lr18 was activated in sterile liquid MRS medium at 37 °C for 24 h, followed by three successive transfers at 1% (*v*/*v*) inoculation into fresh MRS broth and incubation at 37 °C with shaking at 220 rpm for 24 h. Cells were harvested at the exponential phase and suspended in sterile 0.9% NaCl to a density of approximately 10^8^ CFU/mL (OD_600_ = 0.6). Broccoli stalks and florets were washed separately, homogenized with distilled water (1:1, *w*/*w*), and heated in a water bath at 85 °C for 15 min to inactivate endogenous enzymes [20]. An aliquot of the homogenates (30 mL; initial pH 5.75 ± 0.15 for stalks and 6.02 ± 0.24 for florets) was dispensed into 50 mL sterile conical tubes. For fermentation, 5% (*v*/*v*) active Lr18 culture was incubated at 37 °C for 48 h. Samples were freeze-dried or frozen in liquid nitrogen and stored at −80 °C.

### 2.3. Determination of Viable Cell Counts and Physicochemical Properties

#### 2.3.1. Enumeration of Total Viable Counts

Total viable counts were quantified by standard plate count on MRS agar (De Man, Rogosa and Sharpe medium; Beijing Aoboxing Biotech Co., Ltd., Beijing, China) after serial ten-fold dilution in sterile 0.9% NaCl. Plates were incubated anaerobically at 37 °C for 48 h using anaerobic cultivation boxes with AnaeroPack^®^ sachets (Mitsubishi Gas Chemical, Tokyo, Japan) to ensure complete oxygen depletion. Colony enumeration was restricted to plates containing 30–300 colonies, and results were expressed as log CFU/mL.

#### 2.3.2. Physico-Chemical Determination

For total sugar determination, samples were diluted 1:10 (*v*/*v*) with distilled water, heated in a boiling water bath for 10 min, and centrifuged at 8000× *g* for 10 min at 4 °C to collect the supernatant; total sugar content was determined using the anthrone–sulfuric acid method [21], with absorbance measured at 620 nm using a SpectraMax iD5 microplate reader (Molecular Devices, San Jose, CA, USA). For pH, total soluble solids (TSS), and titratable acidity (TA) analysis, samples were centrifuged at 4000× *g* for 10 min at 4 °C before measurement. The pH value was recorded using a PB-10 pH meter (Sartorius, Göttingen, Germany), TSS was determined at 25 °C with a WAY-2S refractometer (Shanghai Precision & Scientific Instrument Co., Ltd., Shanghai, China) and expressed as °Brix, and TA was measured by acid–base titration [2] and calculated as lactic acid equivalents.

### 2.4. Determination of Organic Acids

Organic acids were analyzed by a high-performance liquid chromatography system (HPLC-20AT; Shimadzu, Kyoto, Japan) [22]. Samples were extracted with water, sonicated, and centrifuged at 5000 rpm. The supernatant was filtered and 20 μL was injected onto a C18 column (4.6 × 250 mm, 5 μm, Agilent Technologies, Santa Clara, CA, USA). The mobile phase was 0.01 M potassium dihydrogen phosphate (pH 2.55):methanol (90:10, *v*/*v*) at 1.0 mL/min. Detection was at 210 nm. Organic acids were identified by retention time comparison with standards and quantified using calibration curves.

### 2.5. Quantification of Total Phenolic and Flavonoid Content

Total phenolic content was measured by the Folin–Ciocalteu method [23]. Samples were extracted with 80% methanol (10 mL) and centrifuged at 12,000× *g* for 10 min at 4 °C. The supernatant (1 mL) was mixed with 2.5 mL Folin–Ciocalteu reagent and incubated for 2 min, then 1.5 mL sodium carbonate (75 g/L) was added. After 40 min in the dark, absorbance was measured at 760 nm. Results were expressed as gallic acid equivalents.

Total flavonoid content was determined by the aluminum chloride method [24]. Samples were extracted with 60% ethanol. The filtrate (1 mL) was mixed with 0.5 mL sodium nitrite (5%) for 5 min, then 1 mL aluminum chloride (10%) was added and it was kept in the dark for 5 min. Sodium hydroxide (4 mL, 4%) was added and diluted to 10 mL with 60% ethanol. After 15 min in the dark, absorbance was measured at 510 nm as rutin equivalents.

### 2.6. Volatile Compound Analysis

Volatile compounds were determined by HS-SPME-GC-MS [19]. Samples (2 mL) were placed in 20 mL headspace vials with 40 μL 2-octanol (internal standard, 10,000× dilution), equilibrated at 40 °C for 15 min, and extracted with SPME fiber (Guangzhou Jet Bio-Filtration Co., Ltd., Guangzhou, China) for 30 min. The fiber was desorbed at 250 °C for 5 min. Separation was performed on a DB-WAX column (Agilent Technologies, Santa Clara, CA, USA) with helium carrier (1.0 mL/min). The oven temperature was programmed: 40 °C (2 min), 3 °C/min to 120 °C, and 4 °C/min to 240 °C (3 min). MS was operated in EI mode (70 eV) with full scan (*m*/*z* 30–500) and SIM. Compounds were identified by NIST 12 mass spectral library (NIST, Gaithersburg, MD, USA, 2012) matching (≥80% similarity) and retention indices (<5% error). Quantification was performed by the internal standard method.

### 2.7. Quantification of Vitamin C

Vitamin C was determined by the Fast Blue B salt method [25]. Samples (0.1 g) were extracted with 1 mL cold metaphosphoric acid and centrifuged at 8000× *g* for 20 min at 4 °C. The supernatant (100 μL) was mixed with acetic acid (30 μL), buffer (50 μL), Fast Blue B salt (120 μL), and water (700 μL). After 20 min at 25 °C, absorbance was measured at 420 nm. Results were expressed as mg/100 g fresh weight.

### 2.8. Determination of Tryptophan Metabolites

Tryptophan and related metabolites were analyzed by UPLC-MS/MS (MRM mode) [26]. Separation was performed on a Waters UPLC HSS T3 column (2.1 × 100 mm, 1.8 μm, Waters Corporation, Milford, MA, USA) at 40 °C. The mobile phase was water and acetonitrile (both 0.1% formic acid) at 0.3 mL/min with the following gradients: 0–0.5 min, 1% B; 0.5–5 min, 1–100% B; 5–7 min, 100% B; 7–7.1 min, 100–1% B; and 7.1–9 min, 1% B. Injection volume was 3 μL. MS was operated in ESI+ mode (3.0 kV, 30 V cone voltage, 500 °C desolvation, 1000 L/h). Quantification was performed by external calibration.

### 2.9. Sensory Characteristics Analysis

Sensory properties of fermented broccoli juice were assessed employing a nine-point hedonic scale (Table S1), with slight modifications [27,28]. Sensory evaluation was performed by 9 trained professional panelists (4 males and 5 females, aged 20–35 years old). Five sensory attributes were examined: color, texture, aroma, taste, and overall acceptability. A sensory panel consisting of nine experienced assessors with professional backgrounds in sensory evaluation was recruited from the College of Food Science and Nutritional Engineering, China Agricultural University. All samples were labeled with random three-digit codes and provided to panelists in individual booths under standardized testing conditions. Each attribute was scored on a scale from 0 to 9, where 7–9 indicated excellent quality (e.g., uniform green or yellowish-green color, smooth homogeneous texture, harmonious aroma and flavor), 4–6 represented acceptable quality with minor defects, and 0–3 corresponded to poor quality characterized by severe browning, obvious sedimentation, off-odors, or imbalanced taste. For each sensory attribute, the difference was analyzed by an independent sample *t*-test. The significance level was set at α = 0.05, and *p* > 0.05 was defined as no significant difference (ns).

### 2.10. Statistical Analysis

Data were analyzed using SPSS (Version 21.0). Experiments were performed in triplicate and results expressed as mean ± SD. Normality was tested before ANOVA. Post hoc comparisons used Tukey’s test (equal variance) or Dunnett’s T3 (unequal variance). Significance was set at *p* < 0.05 (highly significant: *p* < 0.01). Principal component analysis (PCA) and volcano plot analysis were performed using R software (version 4.2.0) with the ‘ggplot2’ (v3.4.4), ‘ropls’ (v1.34.0), ‘ggrepel’ (v0.9.3), and ‘pheatmap’ (v1.0.12) packages. Graphs were prepared with GraphPad Prism (Version 10.4.1; GraphPad Software, San Diego, CA, USA).

## 3. Results and Discussion

### 3.1. Physicochemical Properties of Fermented Broccoli Juices

#### 3.1.1. Changes in Total Viable Counts

**Figure 1:**
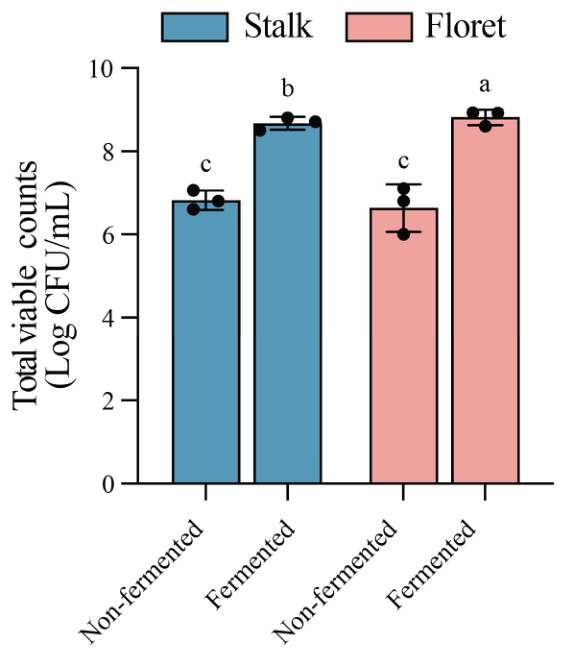
Changes in viable cell counts of broccoli juice before and after LAB fermentation. Error bars indicate the standard deviations from three independent fermentation samples (each sample was collected at three data points). Within the same group, different lowercase superscripts indicate significant differences (*p* < 0.05).

Both broccoli florets and stalks contain carbohydrates including soluble sugars and dietary fiber, which serve as carbon sources for LAB fermentation [29]. As shown in Figure 1, a significant increase in total viable counts was found after fermentation. Compared to non-fermented samples, the viable counts in fermented broccoli stalks and florets increased by 1.85 Log CFU/mL and 2.18 Log CFU/mL, respectively. All samples had total viable counts greater than 8.5 Log CFU/mL after 48 h of fermentation. Therefore, broccoli stalks and florets serve as a valuable source of nutrients for bacterial growth and reproduction.

#### 3.1.2. Changes in Total Soluble Solids (TSS) and Total Sugar

**Figure 2:**
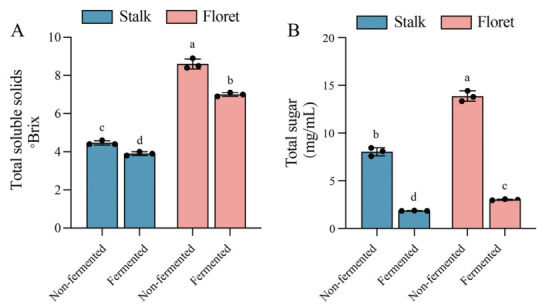
Changes in total soluble solids (**A**) and total sugar (**B**) of broccoli juice before and after LAB fermentation. Error bars indicate the standard deviations from three independent fermentation samples (each sample was collected at three data points). Within the same group, different lowercase superscripts indicate significant differences (*p* < 0.05).

TSS reflect the total dissolved solids in juice, with glucose, fructose, and sucrose accounting for the highest proportions [30]. As shown in Figure 2, the TSS contents of non-fermented stalks and florets were 4.47 °Brix and 8.60 °Brix, and then decreased to 3.90 °Brix and 7.00 °Brix after fermentation, respectively. Similarly, the sugar contents in stalks and florets decreased from 8.03 mg/mL and 13.86 mg/mL to 1.85 mg/mL and 3.02 mg/mL after fermentation, respectively. LAB exhibit a high sugar utilization rate during fermentation. Correlation analysis (Table 1) also indicates that sugar content is strongly positively correlated with total viable count. Compared to non-fermented stalks and florets, the stalks and florets showed similar decreases in sugar content, dropping by 76.95% and 78.18%, respectively. This is in accordance with the previous study on plant-based LAB fermented juice [22]. The greater reduction in TSS reflects high initial sugar availability and microbial metabolic activity. Sugars typically account for a large portion of TSS measurements, but water-soluble components such as organic acids, amino acids, soluble pectin, minerals, vitamin C, and certain phenolic compounds also contribute to the refractive index and are therefore included in the TSS content [31].

#### 3.1.3. Changes in pH, Total Titratable Acids, and Organic Acids

**Figure 3:**
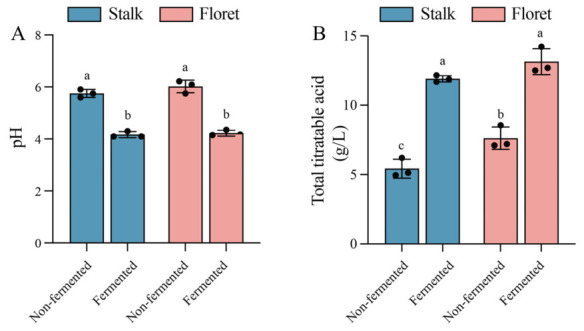
Changes in pH (**A**) and total titratable acids (**B**) of broccoli juice before and after LAB fermentation. Error bars indicate the standard deviations from three independent fermentation samples (each sample was collected at three data points). Within the same group, different lowercase superscripts indicate significant differences (*p* < 0.05).

LAB can utilize carbohydrates and proteins to produce organic acids such as lactic acid, accompanied by a decrease in pH [32,33]. As shown in Figure 3, Lr18 exhibited significant acidification activity in both juices. After fermentation, the pH of broccoli florets and stalks decreased by 1.6 and 1.8 respectively, compared to the non-fermented samples. This showed a negative correlation with the change in total titratable acid contents of both the stalk and floret during the 48 h of fermentation. Compared with the non-fermented stalks and florets, the total titratable acidity of the stalks and florets in the fermentation group increased by 119.34% and 72.44%, respectively. Correlation analysis (Table 1) indicates that a decrease in pH is closely associated with increases in total titratable acidity and total viable count. The titratable acid content increased more significantly in the stalk group, suggesting that a larger proportion of the utilized sugars in the stalk group was converted into titratable organic acids, whereas in the floret group, some sugars were metabolized by microorganisms into other substances [34].

As shown in Table 2, lactic acid was not found in the non-fermented stalk and floret juices, but reached 61.44 ± 4.88 mmol/L and 56.24 ± 2.25 mmol/L after fermentation, respectively. The citric acid in the stalks and florets increased by 2.86 and 2.02 times, whereas malic acid in stalks and florets decreased by 36.7% and 44.9% and oxalic acid decreased by 95.8% and 91.6% after fermentation, respectively. The increase in lactic acid and citric acid was mainly due to the fermentation of sugars by LAB for acid production [35]. In addition, some LAB can, through decarboxylating malic acid into lactic acid, known as malic–lactic fermentation (MLF), result in a decrease in malic acid [36,37]. The decrease in oxalic acid in this study is similar to a previous report that LAB fermentation significantly reduced oxalic acid content in amaranth juice [38]. This may originate from the degradation and utilization of oxalic acid by some strains and the formation of oxalate precipitation from oxalic acid with Ca^2+^ and Mg^2+^, which is carried away during subsequent centrifugation and filtration [39].

### 3.2. Changes in Bioactive Substances

**Figure 4:**
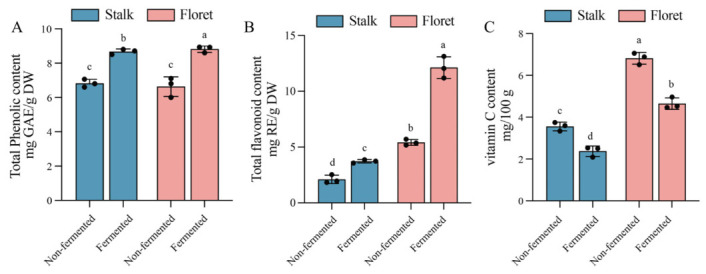
Changes in total phenolic content (**A**), total flavonoid content (**B**), and vitamin C (**C**) of broccoli juice before and after LAB fermentation. Error bars indicate the standard deviations from three independent fermentation samples (each sample was collected at three data points). Within the same group, different lowercase superscripts indicate significant differences (*p* < 0.05).

Polyphenolic compounds, flavonoid content, and vitamin C serve as indicators for evaluating whether fermentation enhances antioxidant activity [39]. As shown in Figure 4A, the total phenolic contents in non-fermented broccoli stalks and florets were 3.37 mg GAE/g and 7.70 mg GAE/g DW, respectively. After 48 h of fermentation, phenolic content in stalks and florets increased to 4.73 mg GAE/g DW and 12.81 mg GAE/g DW. Compared to stalks, fermentation induced greater phenolic production in florets. The increased release of soluble bound and insoluble phenolic compounds from broccoli cell walls contributes to the elevated phenolic content; meanwhile, the deglycosylation of glycosylated phenolics by LAB probably plays an important role in enhancing the total phenolic content [40].

As shown in Figure 4B, the flavonoid content in non-fermented broccoli stalks and florets was 2.18 ± 0.49 mg GAE/g DW and 5.42 ± 0.26 mg GAE/g DW, respectively. As expected, after 48 h of fermentation, these levels increased by 70.94% and 123.66%, respectively, reaching 3.73 ± 0.15 mg GAE/g DW and 12.13 ± 0.97 mg GAE/g DW. These findings are consistent with the previous report about fermented apple juice [41]. The increase results from the enzymatic release of flavonoid aglycones by LAB glycosidases [40]. In addition, bound flavonoids with pectin in the cell walls were more easily dissociated and solubilized during fermentation.

As shown in Figure 4C, vitamin C contents in non-fermented stalks and florets were 3.56 and 6.82 mg/100 g, respectively. After fermentation, both groups showed comparable decreases to 2.37 and 4.65 mg/100 g. Significant vitamin C depletion during fermentation has been observed in the LAB fermentation of fruit and vegetable juices [42,43,44]. This decline is attributed to pro-oxidative conditions such as dissolved oxygen and metal ions, incomplete inactivation of ascorbic acid oxidase, and pH decline during fermentation [42,43,44].

### 3.3. Changes in Tryptophan and Its Metabolites of Broccoli Juice During Fermentation

**Figure 5:**
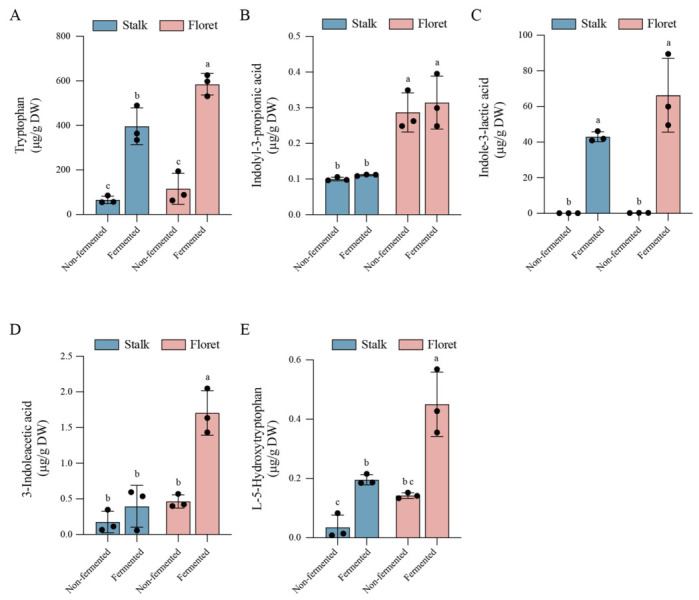
Changes in tryptophan and its metabolites of broccoli juice before and after LAB fermentation. Changes in tryptophan (**A**); indolyl-3-propionic acid (IPA) (**B**); indole-3-lactic acid (ILA) (**C**); 3-indole-acetic acid (IAA) (**D**); L-5-Hydroxytryptophan (5HTP) (**E**). Error bars indicate the standard deviations from three independent fermentation samples (each sample was collected at three data points). Within the same group, different lowercase superscripts indicate significant differences (*p* < 0.05).

It has been reported that proteins are hydrolyzed to release tryptophan (Trp) which can be converted into various indole derivatives such as indole-lactic acid (ILA), indole-acetic acid (IAA), indole-propionic acid (IPA), and L-5-Hydroxytryptophan (5HTP) during LAB fermentation [45]. As shown in Figure 5, the tryptophan contents in unfermented stalks and florets were 65.99 ± 16.89 μg/g DW and 115.50 ± 69.48 μg/g DW, respectively. After 48 h of fermentation, their levels increased by five times and four times, respectively, reaching 396.13 ± 82.33 μg/g DW and 584.85 ± 48.32 μg/g DW. Moreover, there were trace levels of the indole derivatives (ILA, IAA, IPA, and 5HTP) in broccoli stalks and florets, but fermentation induced a significant increase in indole derivatives except IPA. ILA content in stalks and florets reached 43.04 ± 2.76 μg/g DW and 66.33 ± 20.70 μg/g DW, respectively. IAA content in stalks and florets increased to 0.40 ± 0.30 μg/g DW and 1.71 ± 0.31 μg/g DW, respectively. 5HTP content in stalks and florets increased to 0.20 ± 0.02 μg/g DW and 0.45 ± 0.11 μg/g DW, respectively.

In a fermentation system, tryptophan originates not only from the culture medium and the raw materials themselves but may also be released from protein hydrolysis. However, microorganisms only divert a portion of this tryptophan into the indole metabolic pathway. Therefore, a higher residual tryptophan concentration in the final sample typically indicates that the substrate supply capacity exceeds the actual conversion capacity of the indole pathway. Tryptophan-derived indole metabolites exhibit significant microbial specificity. Lactic acid bacteria typically preferentially convert tryptophan to ILA via the aromatic amino acid aminotransferase–indole-lactic acid dehydrogenase pathway, whereas IAA involves a more complex and tightly regulated bypass pathway, resulting in yields that are generally lower than those of ILA. IPA, on the other hand, primarily relies on the flavin reductase/FMN biosynthesis cluster-related reduction pathway common in anaerobic bacteria and is typically present at extremely low levels or absent in lactic acid bacteria [46]. Consequently, in lactic acid bacteria fermentation systems, tryptophan is usually the highest, followed by ILA, then IAA, with IPA being the lowest. The ability of bacteria to naturally produce 5-HTP is not widespread [47]. In particular, LAB generally exhibit weak 5-HTP-producing capacity. Consequently, 5-HTP contents remained at low levels across all groups.

### 3.4. Changes in Volatile Organic Compounds (VOCs) of Broccoli Juice

**Figure 6:**
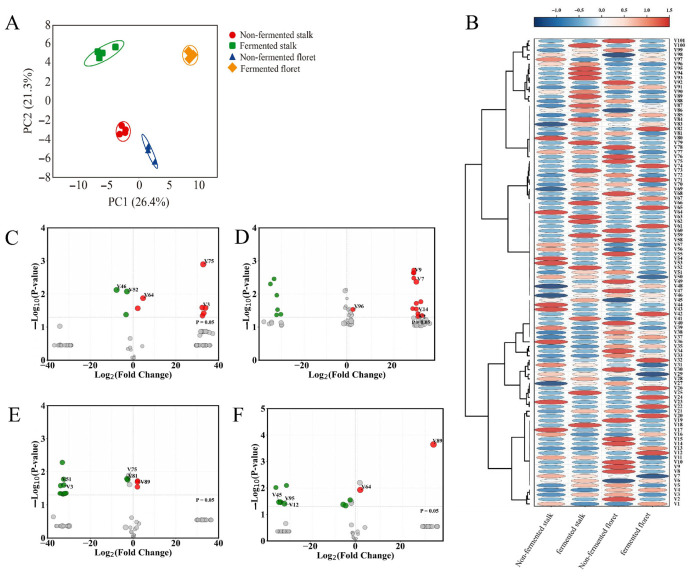
Changes in volatile organic compounds (VOCs) of broccoli juice. Principal component analysis of broccoli stalks and florets before and after fermentation (**A**); the heatmap of broccoli stalks and florets before and after fermentation (**B**); volcano plot of differential flavor compounds in broccoli stalks and florets before fermentation (**C**); volcano plot of differential flavor compounds in broccoli stalks before and after fermentation (**D**); volcano plot of differential flavor compounds in broccoli florets before and after fermentation (**E**); volcano plot of differential flavor compounds in broccoli stalks and florets after fermentation (**F**). In panels (**C**–**F**), red and green dots indicate compounds with log_2_FC > 1 and log_2_FC < −1, respectively (VIP > 1.0, *p* < 0.05).

Flavor compounds in broccoli stalks and florets before and after fermentation were identified via HS-SPME-GC-MS analysis (Figure 6 and Tables S2–S6). A total of 101 volatile compounds were identified, classified into ten major groups: esters (15 compounds), amides (2), hydrocarbons (15), ketones (7), acids (8), oxygenated compounds (23), aldehydes (7), ethers (2), sulfur-containing compounds (7), and alcohols (15). In terms of compound distribution, oxygenated compounds, hydrocarbons, esters, and alcohols constituted the primary components.

Regarding the general distribution of aroma characteristics, as presented in Figure 6C, pentanenitrile, 5-(methylthio)-(V46, log_2_FC = −7.71), which exhibits sulfur and garlic notes; benzaldehyde (V52, log_2_FC = −2.84), which has an almond aroma; and heptanal (V95, log_2_FC = −3.27), which has a fatty aroma were significantly more abundant in non-fermented broccoli stalks than in the florets. The floret tends to accumulate compounds with floral, fruity, and green aromas, such as 2,4-heptadienal, (E,E)-(V3, log_2_FC = 32.38) and 2-hexen-1-ol, (E)-(V64, log_2_FC = 32.82). This not only reflects tissue-specificity in secondary metabolism but also reveals tissue-specific mechanisms underlying flavor formation in broccoli stalks and florets. Hong et al. [48] indicate that the stalks have a milder, sweeter flavor, and that the typical cruciferous pungency and bitterness are generally less pronounced than in the flower florets, which differs from the findings of this study. A previous study showed that glucosinolates may be translocated from the florets to the stalks during postharvest storage, making the stalks more likely to accumulate irritant precursors, and thus more biased toward pungent, stimulating flavors [49]. Myrosinase hydrolyzes glucosinolates to form an unstable intermediate, which subsequently undergoes rearrangement or shunting to generate volatile flavor compounds such as thiocyanates, isothiocyanates, and nitriles [50].

Through volcano plot analysis (Figure 6D,E) and differential screening, a total of 19 significantly different volatile compounds were detected in broccoli stalks before and after fermentation, and 17 significantly different volatile compounds were detected in broccoli florets before and after fermentation (*p* < 0.05, VIP > 1.0, |log2FC| > 1). These differentially expressed compounds primarily encompass multiple categories including alcohols, ketones, esters, aldehydes, nitrogen-containing compounds, and sulfur-containing compounds, reflecting the multidimensional regulatory effects of fermentation on the volatile components of broccoli stalks and florets.

In non-fermented broccoli stalks, C6 unsaturated aldehydes represented by (E)-2-hexenal (V63, log_2_FC = −32.29) exhibited the characteristic grassy, green, leafy top note aroma. During fermentation, LAB converted this aldehyde into corresponding alcohols through aldehyde reduction [51]. Simultaneously, the CO_2_ produced by fermentation resulted in the loss of aeration of this top flavor component, which only originated from the raw material and was not produced subsequently, leading to a significant decrease in its content [52]. Certain original esters, such as 1-butanol, 2-methyl-, acetate (V22, log_2_FC = −33.92), undergo hydrolysis under acidic conditions and esterase action, leading to reduced levels [53]. Conversely, the compounds that showed an increase were predominantly alcohols, esters and ketones. Examples of this reduction trend, which involved decreasing aldehydes while increasing alcohols, included 1-hexanol (V96, log_2_FC = 2.72) and 1-penten-3-ol (V14, log_2_FC = 31.5). This resulted in a softer green character [54]. Pentanoic acid, 3-methyl-, methyl ester (V2, log_2_FC = 31.62) enhanced sweet fruit notes and roundness. Cyclic and unsaturated ketones, such as 2-cyclopenten-1-one, 3-ethyl-(V36, log_2_FC = 29.73) and 3,5-octadien-2-one, (E,E)-(V30, log_2_FC = 30.85), impart ripened, nutty, or fermented characteristics [55,56]. Overall, fermentation diminished the fresh, green, and pungent compounds associated with stalks while amplifying the complexity and ripe fruit aromas derived from the fermentation process.

During the fermentation of broccoli florets, acetic acid (VF, log_2_FC = 2.04) exhibited an upward trend. As a typical metabolite of LAB (particularly those following heterofermentative pathways), it directly enhances sourness and pungent notes [57]. Methanethiol (V70, log_2_FC = 2.11), an ultra-low-threshold sulfur compound, imparts onion–garlic and fermented sulfur notes. Its increase contributes to an enhanced flavor profile and complexity. The rise in 4-Hexen-1-ol, acetate (V89, log_2_FC = 2.09) reflects the partial esterification of hexen-1-ol, shifting toward softer fruity aromas. Conversely, declining compounds predominantly represent raw top note aromas of fresh produce, such as hexanal (V97, log_2_FC = −33.84), (Z)-2-penten-1-ol (V81, log_2_FC = −2.53), and 1-penten-3-ol (V30, log_2_FC = −32.70), which primarily contribute grassy, green, and mushroom-like aromas [51]. These C5/C6 aldehydes and alcohols are easily converted during fermentation acidification and microbial reductive metabolism or are lost through CO_2_ stripping. The decrease in green leaf esters like (Z)-3-hexen-1-yl acetate (V92, log_2_FC = −31.88) significantly diminishes the inherent freshness of vegetables. Decreases in lipid oxidation-related compounds like 2,4-heptadienal (V3, log_2_FC = −32.38) and 3,5-octadien-2-one (V1, log_2_FC = −32.49) reduce greasy, rancid, or oxidized odors. The decrease in methyl thiocyanate (V68, log_2_FC = −33.08) and certain nitriles and heterocyclic compounds helps mitigate pungent or potential off flavors [58].

Overall, LAB fermentation of broccoli florets emphasizes enhanced acid and sulfur notes while reducing various green leaf esters and fruity esters, resulting in a flavor profile that leans acidic, fermented, and even slightly pungent. Both stalks and florets share a common characteristic. Volatile compounds associated with fresh produce lipids and green leaf pathways (C5 and C6 aldehydes, alcohols, green leaf-related unsaturated compounds, etc.) decrease or undergo alteration after fermentation. The fermentation process (acidification, reducing environment, metabolic conversion, CO_2_ stripping) generally diminishes the freshly cut green and crisp sensation. Furthermore, from an aromatic profile perspective, there were differences between the samples. Fermentation optimized the overall flavor characteristics of broccoli stalks. For samples of the florets, which were rich in fruity lipid flavor compounds, fermentation reduced their fruitiness while increasing acidity and sulfur notes. In contrast, for stalk samples with fewer fruity flavor compounds but more pungent odor compounds, fermentation enhanced fruitiness and roundness while reducing pungency.

Analysis of differences between fermented broccoli stalks and florets revealed that while the stalk group exhibited reduced levels of pungent flavor compounds during fermentation, it still displayed sharp acidity and a robust flavor profile (Figure 6F). The difference from fermented florets lies in the enrichment of multiple nitriles, including sulfur-containing nitriles and epithionitriles characteristic of the cruciferous defense cleavage pathway, such as hexanenitrile, 5-methyl-(V45, log_2_FC = −33.80); heptanonitrile (V95, log_2_FC = −33.15); benzenepropanenitrile (V52, log_2_FC = −4.26); pentanenenitrile, 5-(methylthio)-(V46, log_2_FC = −5.35); and cyano-3,4-epithiobutane (V12, log_2_FC = −31.43). These compounds are more likely to impart pungent, sharp, and unpleasant flavors in sensory evaluations. Among these, 1-cyano-3,4-epithiobutane has been explicitly reported as a key epithionitrile formed during the hydrolysis of cruciferous glucosinolates. It is not a product of the atypical sweet fruit aroma pathway but rather contributes to vegetable-like and pungent sulfur characteristics [59]. Concurrently, methyltartronic acid (V25, log_2_FC = −35.24), belonging to the polycarboxylic acids (contributing more to acidity than primary aroma), can be understood as one factor contributing to the overall sharper acidity and astringency [60]. Conversely, floret fermentation yields higher levels of green leaf volatiles (GLVs) and their acetate esters, such as (E)-2-Hexen-1-ol (V64, log_2_FC = 2.20) and 4-Hexen-1-ol acetate (V89, log_2_FC = 34.74), which typically impart fresher grassy, green fruit, slightly sweet fruity aromas, and softer, rounder fruity flavors.

### 3.5. Sensory Evaluation

**Figure 7:**
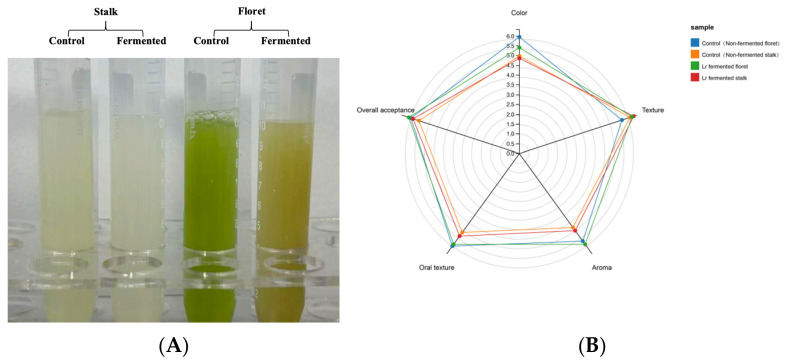
Changes in sensory scores (**A**) and appearance (**B**) of broccoli juice before and after fermentation.

The human taste and olfactory systems play a crucial role in sensory perception. The sensory scores for each group of samples are shown in Figure 7 and Table S7. In the non-fermented samples, all sensory attributes of the florets were slightly superior to those of the stalks. Fermentation reduced the color scores of all samples (no significant differences). Before fermentation, the sensory scores for aroma, texture, and overall acceptability were lower for both the stalks and the florets. Fermentation slightly improved the sensory quality of both the stalks and the florets (no significant differences). After fermentation, the florets exhibited the highest overall acceptability, while the stalks had the best texture, which is consistent with the results for volatile compounds. Similarly, studies have found that fermentation also improves the sensory ratings of *Brassica rapa* L. juice [61].

## 4. Conclusions

This study demonstrated that fermentation with Lr18 significantly altered the physicochemical, nutritional, and flavor properties of broccoli stalk and floret juices. Fermentation increased total phenolic and flavonoid content, reduced oxalic acid, and converted malic acid to lactic acid. Vitamin C decreased, while tryptophan-derived indole metabolites (ILA, IAA) accumulated. Flavor analysis revealed tissue-specific responses: fermentation reduced pungency and enhanced fruitiness in stalks, whereas it increased acidity and sulfur notes in florets. Sensory evaluations indicate that fermentation improved the sensory quality of the juice from the florets and stalks of broccoli. Broccoli stalks, often discarded as waste, exhibited comparable or superior organic acid profiles after fermentation. In particular, the successful utilization of broccoli may provide a cost-effective approach for reducing raw material waste and creating value-added products in the vegetable processing industry. Future studies should further evaluate process scalability, storage stability, consumer acceptance, and the optimization of fermentation conditions to better balance nutritional retention and flavor improvement. In addition, exploring mixed starter cultures or strain-specific fermentation strategies may help to tailor sensory profiles and expand the commercial applications of fermented broccoli-based beverages.

## Figures and Tables

**Table 1:** Correlation analysis of indicators in broccoli juice before and after fermentation.

Indicators	Total Bacteria	pH	Total Titratable Acid	Total Soluble Solids	Total Sugar
Total bacteria	1	−0.891 **	0.800 **	−0.279	−0.820 **
pH	−0.891 **	1	−0.825 **	0.197	0.844 **
Total titratable acid	0.800 **	−0.825 **	1	−0.200	−0.744 **
Total soluble solids	−0.279	0.197	−0.200	1	0.22
Total sugar	−0.820 **	0.844 **	−0.744 **	0.22	1

Note: The values in the table are Pearson correlation coefficients (r); ** indicates *p* < 0.01, representing significant differences; mean represents the mean value of each indicator and variance represents the variance of each indicator. The matrix was calculated based on nine valid samples.

**Table 2:** Content of four organic acids in broccoli juice before and after fermentation.

Content	Stalk		Floret	
(mmol/L)	Non-Fermented	Fermented	Control	Non-Fermented
Lactic acid	ND	61.44 ± 4.88 ^a^	ND	56.24 ± 2.25 ^a^
Malic acid	12.28 ± 2.79 ^a^	7.77 ± 0.61 ^b^	7.62 ± 0.59 ^a^	4.20 ± 0.42 ^b^
Citric acid	3.10 ± 0.11 ^a^	11.97 ± 1.69 ^b^	4.92 ± 0.03 ^a^	14.88 ± 1.57 ^b^
Oxalic acid	3.58 ± 0.28 ^a^	0.15 ± 0.02 ^b^	2.27 ± 0.32 ^a^	0.19 ± 0.02 ^b^

Note: Data are expressed as mean ± standard deviation (SD) of three independent replicates. For each organic acid, values were analyzed separately for stalks and florets by one-way ANOVA followed by post hoc pairwise comparisons. Different lowercase letters within the same row and tissue indicate significant differences between control and fermented groups at *p* < 0.05. ND, not detected.

## Data Availability

The original contributions presented in this study are included in the article/Supplementary Materials. Further inquiries can be directed to the corresponding authors.

## Supplementary Materials

The following supporting information can be downloaded at https://www.mdpi.com/article/10.3390/foods15091519/s1: Table S1: Sensory evaluation standards. Table S2: The relative contents of volatile compounds in non-fermented and Lr fermented broccoli (stalk and floret) by HS-SPME-GC-MS. Table S3: Screening of characteristic volatile components in broccoli florets and stalks. Table S4: Screening of characteristic volatile components in non-fermented and Lr fermented broccoli stalks. Table S5: Screening of characteristic volatile components in non-fermented and Lr fermented broccoli florets. Table S6: Screening of characteristic volatile components in fermented broccoli florets and stalks. Table S7: Sensory evaluation scores of broccoli juices before and after fermentation.

## Abbreviations

The following abbreviations are used in this manuscript:

Lr 18	*Limosilactobacillus reuteri* 18
LAB	Lactic acid bacteria
GABA	γ-aminobutyric acid
HS-SPME-GC-MS	Headspace Solid-Phase Microextraction–Gas Chromatography–Mass Spectrometry
ILA	Indole-3-lactic acid
TSS	Total soluble solids
IPA	Indolyl-3-propionic acid
IAA	3-Indoleacetic acid
5HTP	L-5-Hydroxytryptophan
VOCs	Volatile organic compounds

## References

[1] Amicarelli V., Lagioia G., Bux C. (2021). Global warming potential of food waste through the life cycle assessment: An analytical review. Environ. Impact Assess. Rev..

[2] Višnjevec A.M., Barp L., Fanesi B., Iacumin L., Lucci P., Moret S. (2025). Phenolic compounds and glucosinolates composition of cauliflower and broccoli byproducts puree after the lactic acid fermentation. Food Chem..

[3] Mallhi I.Y., Javed F., Hameed S., Tufail T., Bader Ul Ain H., Tufail T., Saeed F., Ansar Rasul Suleria H. (2026). Broccoli and Its By-products: Turning Waste into Wellness. Importance of Plant Based Byproducts: Nutritional and Functional Properties.

[4] Gudiño I., Casquete R., Martín A., Wu Y., Benito M.J. (2024). Comprehensive Analysis of Bioactive Compounds, Functional Properties, and Applications of Broccoli By-Products. Foods.

[5] Cano-Gonzalez C.N., Aguirre-Loredo R.Y., Aguilar C.N., Rodriguez-Herrera R., Soriano-Melgar L.d.A.A. (2025). Microwave-assisted extraction of phenolic compounds from broccoli waste with antioxidant and antihyperglycemic activities: A sustainable approach. Bioresour. Technol. Rep..

[6] Wang Q., Zhao Y., Zhang W., Deng J., Yang H. (2025). Valorization of broccoli waste: Unlocking its potential as a functional food ingredient for sustainable nutrition. J. Adv. Res..

[7] Hwang J.H., Lim S.B. (2015). Antioxidant and anticancer activities of broccoli by-products from different cultivars and maturity stages at harvest. Prev. Nutr. Food Sci..

[8] Alvarez-Jubete L., Valverde J., Kehoe K., Reilly K., Rai D.K., Barry-Ryan C. (2014). Development of a Novel Functional Soup Rich in Bioactive Sulforaphane Using Broccoli (*Brassica oleracea* L. ssp. *italica*) Florets and Byproducts. Food Bioprocess Technol..

[9] Liu M., Zhang L., Ser S.L., Cumming J.R., Ku K.-M. (2018). Comparative Phytonutrient Analysis of Broccoli By-Products: The Potentials for Broccoli By-Product Utilization. Molecules.

[10] Huang R., Fang Y., Zhong Y., Wang D., Lu W., Zhao H., Deng Y. (2026). Advancing fermentation science: Microbial dynamics, metabolomics, and safety in fermented vegetables. J. Future Foods.

[11] de Wolf R.X.M., Hider R.N., Breitmeyer J., Serventi L. (2025). Effect of lactic fermentation and matrix on phenolic content, bioaccessibility, and scavenging activity of beetroot beverages. Eur. Food Res. Technol..

[12] Szutowska J., Gwiazdowska D., Rybicka I., Pawlak-Lemańska K., Biegańska-Marecik R., Gliszczyńska-Świgło A. (2021). Controlled fermentation of curly kale juice with the use of autochthonous starter cultures. Food Res. Int..

[13] Ciska E., Honke J., Drabińska N. (2021). Changes in glucosinolates and their breakdown products during the fermentation of cabbage and prolonged storage of sauerkraut: Focus on sauerkraut juice. Food Chem..

[14] Cruz-Casas D.E., Ramos-González R., Prado-Barragán L.A., Iliná A., Aguilar C.N., Rodríguez-Herrera R., Tsopmo A., Flores-Gallegos A.C. (2025). Protein hydrolysates with ACE-I inhibitory activity from amaranth seeds fermented with *Enterococcus faecium*-LR9: Identification of peptides and molecular docking. Food Chem..

[15] Li Y., He W., He Y., Liu S., Hu X., Bian S., Song X., Yin J., Nie S., Xie M. (2025). Fermentation of celery (*Apium graveolens* L.) with *Lactobacillus plantarum* NCU116: Impact on physicochemical properties, free amino acids, and volatile aroma compounds. Food Biosci..

[16] Aihaiti A., Zhao L., Maimaitiyiming R., Wang L., Liu R., Mu Y., Chen K., Wang Y. (2025). Changes in volatile flavors during the fermentation of tomato (*Solanum lycopersicum* L.) juice and its storage stabilization. Food Chem..

[17] Marco M.L., Cunningham M., Bischoff S.C., Clarke G., Delzenne N., Lewis J.D., Meisel M., Merenstein D., O’Toole P.W., Staudacher H.M. (2026). The International Scientific Association for Probiotics and Prebiotics (ISAPP) consensus statement on the definition and scope of gut health. Nat. Rev. Gastroenterol. Hepatol..

[18] Tripathi M.K., Giri S.K. (2014). Probiotic functional foods: Survival of probiotics during processing and storage. J. Funct. Foods.

[19] Xu X., Bi S., Lao F., Chen F., Liao X., Wu J. (2021). Comprehensive investigation on volatile and non-volatile metabolites in broccoli juices fermented by animal- and plant-derived *Pediococcus pentosaceus*. Food Chem..

[20] Munyaka A.W., Makule E.E., Oey I., Van Loey A., Hendrickx M. (2010). Thermal stability of L-ascorbic acid and ascorbic acid oxidase in broccoli (*Brassica oleracea* var. *italica*). J. Food Sci..

[21] Li J., Zhao W., Pan X., Lao F., Liao X., Shi Y., Wu J. (2022). Improvement of antioxidant properties of jujube puree by biotransformation of polyphenols via *Streptococcus thermophilus* fermentation. Food Chem. X.

[22] Yang L., Zhao Y., Zhou Y., Zhao Q., Yuan S., Ma C., Dong L., Luo Y., Hu X., Chen F. (2025). Study on Physicochemical Properties, Antioxidant Activity and Flavor Quality in the Fermentation of a Plant-Based Beverage by Different Lactic Acid Bacteria. Foods.

[23] Martín-Gómez J., García-Martínez T., Varo M.Á., Mérida J., Serratosa M.P. (2023). Enhance Wine Production Potential by Using Fresh and Dried Red Grape and Blueberry Mixtures with Different Yeast Strains for Fermentation. Foods.

[24] Martínez S., Fuentes C., Carballo J. (2022). Antioxidant Activity, Total Phenolic Content and Total Flavonoid Content in Sweet Chestnut (*Castanea sativa* Mill.) Cultivars Grown in Northwest Spain under Different Environmental Conditions. Foods.

[25] Lester G.E., Lewers K.S., Medina M.B., Saftner R.A. (2012). Comparative analysis of strawberry total phenolics via Fast Blue BB vs. Folin–Ciocalteu: Assay interference by ascorbic acid. J. Food Compos. Anal..

[26] Yılmaz C., Gökmen V. (2018). Determination of tryptophan derivatives in kynurenine pathway in fermented foods using liquid chromatography tandem mass spectrometry. Food Chem..

[27] Wang S., Hu A., Wu W., Yuan H., Li X., Muratkhan M., Wang Y., Ma H., Wang X., Lü X. (2025). Sensory improvement of fermented apple juice diluted from concentrate by lactic acid bacteria. J. Food Sci..

[28] Jiang Y.-H., Li Y.-Y., Zhao Y.-T., Hu Q.-Y., Zhou Y.-X., Zheng Y., Gao Y.-Y., Ibrahim A.A., Xin W.-G., Suo H.-Y. (2026). Insights into changes in functional properties of strawberry juice induced by different types of probiotic fermentation: From molecular to regulatory mechanisms. Food Chem. X.

[29] Zhao Y., Zhang Y., Yang H., Xu Z., Li Z., Zhang Z., Zhang W., Deng J. (2024). A comparative metabolomics analysis of phytochemcials and antioxidant activity between broccoli floret and by-products (leaves and stalks). Food Chem..

[30] Gong Z., Zhi Z., Zhang C., Cao D. (2025). Non-Destructive Detection of Soluble Solids Content in Fruits: A Review. Chemistry.

[31] Wu B., Liu J., Yang W., Zhang Q., Yang Z., Liu H., Lv Z., Zhang C., Jiao Z. (2021). Nutritional and flavor properties of grape juice as affected by fermentation with lactic acid bacteria. Int. J. Food Prop..

[32] Jiang K.-L., Liu L., Pan W.-J. (2025). Two lactic acid bacteria strains isolated from naturally fermented foods improves physicochemical quality, antioxidant capacity, shelf life stability and metabolic profiles of Dangshan pear (*Pyrus* spp.) juice. Food Res. Int..

[33] Liao Y., Cheng Y., Zheng J., Li Z., Wang F., Li L. (2025). ·Lactic Acid Bacteria Fermentation Advances in Fruit-Vegetables: Enhancing Quality and Bioactivity. Food Bioprocess Technol..

[34] Bangar S.P., Suri S., Trif M., Ozogul F. (2022). Organic acids production from lactic acid bacteria: A preservation approach. Food Biosci..

[35] Li X., Li Y., Gao J., Mi S., Mao K., Zhang T., Wang X., Sang Y. (2023). Chemical composition of naturally-fermented mixed fruit product and in vitro bioactivities. LWT.

[36] Hu L., Chen X., Cao Y., Gao P., Xu T., Xiong D., Zhao Z. (2024). *Lactiplantibacillus plantarum* exerts strain-specific effects on malolactic fermentation, antioxidant activity, and aroma profile of apple cider. Food Chem. X.

[37] Bergentall M.K., Malafronte L., As D., Calmet E., Melin P. (2024). Reduction of malic acid in bilberry juice by *Lactiplantibacillus plantarum*-mediated malolactic fermentation. Eur. Food Res. Technol..

[38] Filannino P., Di Cagno R., Trani A., Cantatore V., Gambacorta G., Gobbetti M. (2017). Lactic acid fermentation enriches the profile of biogenic compounds and enhances the functional features of common purslane (*Portulaca oleracea* L.). J. Funct. Foods.

[39] Dissanayake I.H., Tabassum W., Alsherbiny M., Chang D., Li C.G., Bhuyan D.J. (2025). Lactic acid bacterial fermentation as a biotransformation strategy to enhance the bioavailability of phenolic antioxidants in fruits and vegetables: A comprehensive review. Food Res. Int..

[40] Gaur G., Gänzle M.G. (2023). Conversion of (poly)phenolic compounds in food fermentations by lactic acid bacteria: Novel insights into metabolic pathways and functional metabolites. Curr. Res. Food Sci..

[41] Li H., Huang J., Wang Y., Wang X., Ren Y., Yue T., Wang Z., Gao Z. (2021). Study on the nutritional characteristics and antioxidant activity of dealcoholized sequentially fermented apple juice with *Saccharomyces cerevisiae* and *Lactobacillus plantarum* fermentation. Food Chem..

[42] Zeng H., Shuai Y., Zeng X., Xin B., Huang M., Li B., Qiao J., Wang Y., Qiu X., Wang C. (2021). Evaluation of health-related composition and bioactivity of five fruit juices following *Lactobacillus plantarum* fermentation and simulated digestion. Int. J. Food Sci. Technol..

[43] Tong C., Chen X., Deng R., Gao H. (2025). Dynamic changes in physicochemical characteristics, bioactivity and flavor profile of fermented strawberry juice by Lactiplantibacillus plantarum. Food Chem..

[44] Cele N.P., Akinola S.A., Manhivi V.E., Shoko T., Remize F., Sivakumar D. (2022). Influence of Lactic Acid Bacterium Strains on Changes in Quality, Functional Compounds and Volatile Compounds of Mango Juice from Different Cultivars during Fermentation. Foods.

[45] Savijoki K., Ingmer H., Varmanen P. (2006). Proteolytic systems of lactic acid bacteria. Appl. Microbiol. Biotechnol..

[46] Roager H.M., Licht T.R. (2018). Microbial tryptophan catabolites in health and disease. Nat. Commun..

[47] Shi X., Zhao G., Li H., Zhao Z., Li W., Wu M., Du Y.-L. (2023). Hydroxytryptophan biosynthesis by a family of heme-dependent enzymes in bacteria. Nat. Chem. Biol..

[48] Hong S.J., Jeong H., Yoon S., Jo S.M., Lee Y., Park S.-S., Shin E.-C. (2023). Evaluation of taste and aroma compounds in oven-roasted broccoli floret and stem as affected by different times using electronic tongue and electronic nose. Int. J. Food Sci. Technol..

[49] Casajús V., Howe K., Fish T., Civello P., Thannhauser T., Li L., Lobato M.G., Martínez G. (2023). Evidence of glucosinolates translocation from inflorescences to stems during postharvest storage of broccoli. Plant Physiol. Biochem..

[50] Andernach L., Schury C., Nickel M., Böttger J., Kaufmann M., Rohn S., Granvogl M., Hanschen F.S. (2024). Non-enzymatic degradation of aliphatic Brassicaceae isothiocyanates during aqueous heat treatment. Food Chem..

[51] Molina G.E.S., Ras G., da Silva D.F., Duedahl-Olesen L., Hansen E.B., Bang-Berthelsen C.H. (2025). Metabolic insights of lactic acid bacteria in reducing off-flavors and antinutrients in plant-based fermented dairy alternatives. Compr. Rev. Food Sci. Food Saf..

[52] Haefliger O.P., Jeckelmann N. (2013). Stripping of aroma compounds during beer fermentation monitored in real-time using an automatic cryotrapping sampling system and fast gas chromatography/mass spectrometry. Anal. Methods.

[53] Akram F., Fatima T., Shabbir I., Haq I.u., Ibrar R., Mukhtar H. (2025). Abridgement of Microbial Esterases and Their Eminent Industrial Endeavors. Mol. Biotechnol..

[54] Fauconnier M.L., Mpambara A., Delcarte J., Jacques P., Thonart P., Marlier M. (1999). Conversion of green note aldehydes into alcohols by yeast alcohol dehydrogenase. Biotechnol. Lett..

[55] Rajendran S., Silcock P., Bremer P. (2023). Flavour Volatiles of Fermented Vegetable and Fruit Substrates: A Review. Molecules.

[56] Yu A.-N., Zhang A.-D. (2010). The effect of pH on the formation of aroma compounds produced by heating a model system containing L-ascorbic acid with L-threonine/L-serine. Food Chem..

[57] Hao Y., Kang J., Guo Y., Meng L., Li Z., Qin X. (2025). Bacterial interactions mediated by acetic acid and their impact on flavor profile during acetic acid fermentation stage of Shanxi aged vinegar. Food Biosci..

[58] Zheng C., Yang Y., Wei F., Lv X., Xia Z., Qi M., Zhou Q. (2023). Widely targeted metabolomics reveal the glucosinolate profile and odor-active compounds in flowering Chinese cabbage powder. Food Res. Int..

[59] Hanschen F.S., Kaufmann M., Kupke F., Hackl T., Kroh L.W., Rohn S., Schreiner M. (2018). Brassica vegetables as sources of epithionitriles: Novel secondary products formed during cooking. Food Chem..

[60] Yan Y., Zou M., Tang C., Ao H., He L., Qiu S., Li C. (2024). The insights into sour flavor and organic acids in alcoholic beverages. Food Chem..

[61] Qin L., Zheng J., Fan B., Zhou Y., Zhu J., Sun J., Li J., Wang F., Liu J. (2025). Co-fermentation with lactic acid bacteria and prune puree modulates volatile profile and bioactivity of Qiamagu juice. Food Res. Int..

