# Supplementary material for: Comparative Analysis of Quality Attributes and Flavor Profiles of Broccoli (Brassica oleracea var. italica) Stalk and Floret Juices Fermented by Limosilactobacillus reuteri
Source: Foods. 2026 Apr 27;15(9):1519. doi: 10.3390/foods15091519 (PMC13164264; doi:10.3390/foods15091519)
Supplement: Supplementary file 1 [file foods-15-01519-s001.zip › foods-4208556-supplementary.pdf]

Supplementary Data

Table Legends

- **Table S1** Sensory evaluation standards.
- **Table S2** The relative contents of volatile compounds in non-fermented and Lr fermented broccoli (stalk and floret) by HS-SPME-GC-MS.
- **Table S3** Screening of characteristic volatile components in broccoli florets and stalks.
- **Table S4** Screening of characteristic volatile components in non-fermented and Lr fermented broccoli stalks.
- **Table S5** Screening of characteristic volatile components in non-fermented and Lr fermented broccoli florets.
- **Table S6** Screening of characteristic volatile components in fermented broccoli florets and stalks.
- **Table S7** Sensory evaluation scores of broccoli juices before and after fermentation.

Table S1. Sensory evaluation standards.

Sensory index	Scoring criteria	Score
Color	Uniform green or yellowish-green color, normal and attractive appearance	7-9
	Uneven color, without obvious browning	4-6
	Abnormal color	0-3
Texture	Homogeneous and smooth, good flowability, non-sticky	7-9
	Relatively uniform, moderate flowability	4-6
	Obvious sedimentation and stratification, coarse texture with agglomeration	0-3
Aroma	Harmonious aroma with prominent fresh broccoli notes and moderate fermented lactic acid fragrance, free of off-odors	7-9
	Mild aroma, slightly pronounced fresh or lactic acid notes, no off-odors	4-6
	Insufficient or abnormal aroma, dominant earthy notes, excessive sourness, or obvious off-odors	0-3
Taste	Delicate and refreshing mouthfeel, well-balanced sweet-sour profile, free from astringency, bitterness, or pungency	7-9

	Acceptable mouthfeel, slightly excessive sourness or sweetness	4-6
	Rough mouthfeel, extremely imbalanced taste (overly sour, bitter or astringent)	0-3
Overall acceptability	Excellent overall quality with good coordination among color, aroma, taste and texture	7-9
	Acceptable overall quality with minor imperfections	4-6
	Poor overall quality with obvious sensory defects	0-3

Table S2. The relative contents of volatile compounds in non-fermented and Lr fermented broccoli (stalk and floret) by HS-SPME-GC-MS.

No.	Name	CAS	Control (Non-fermented stalk)	Lr fermented stalk	Control (Non-fermented floret)	Lr fermented floret	Control (Non-fermented stalk) VS Lr fermented stalk	Control (Non-fermented floret) VS Lr fermented floret	Category	Aroma
V2	Methyl 3-methylpentanoate	2177-78-8	ND	1.32±0.15	ND	ND	***		Ester	Fruity, sweet
V4	(E,Z)-3-Hexenyl 2-butenoate	65405-80-3	3.60±4.77	5.42±0.15	ND	2.75±0.15	*	***	Ester	Fruity, leafy green
V5	Ethyl N-propargyloxycarbonyl-L-alaninate	1000322-67-3	49.36±0.15	ND	ND	3.61±0.15	***	***	Ester	Pungent, ammonia-like
V10	3-Hydroxyphenyl benzoate	136-36-7	ND	ND	0.40±0.15	ND		***	Ester	Phenolic
V17	2,3-Butanediol dinitrate	6423-45-6	4.01±0.15	4.22±0.15	2.88±0.34	2.15±0.32	NS	*	Ester	Sweet, fruity
V22	2-Methylbutyl acetate	624-41-9	6.48±0.15	ND	ND	ND	***		Ester	Fruity, sweet
V26	Methyl 2-oxobutanoate	3952-66-7	ND	ND	0.47±0.15	ND		***	Ester	Ester, fruity
V68	Hexanoic acid, methyl ester	106-70-7	ND	ND	1.21±0.39	ND		***	Ester	Fruity, sweet
V7	Hexyl formate	629-33-4	ND	ND	7.51±0.1	ND		***	Ester	Fruity,

2					5					sweet
V73	(Z)-3-Hexen-1-yl formate	33467-73-1	4.80±0.15	5.39±0.15	3.74±0.15	ND	NS	***	Ester	Green, fruity
V75	Methyl thiocyanate	556-64-9	ND	0.77±0.15	0.76±0.27	0.33±0.15	***	*	Ester	Sulfurous
V89	4-Hexen-1-yl acetate	72237-36-6	ND	0	2.71±0.15	2.87±0.63		NS	Ester	Fruity, floral
V91	Acetic acid, pentyl ester	628-63-7	6.11±3.31	3.92±1.68	1.80±0.20	1.71±0.15	*	NS	Ester	Fruity, sweet
V92	(Z)-3-Hexen-1-yl acetate	3681-71-8	ND	ND	0.79±0.15	ND		***	Ester	Green, fruity
V100	Butyl cyclobutyl oxalate	1000309-69-4	ND	ND	21.58±0.15	ND		***	Ester	Ester, fruity
V48	N-Methylformamide	123-39-7	0.20±0.15	ND	ND	ND	***		Amide	Amine-like, fishy
V74	Formamide	27735	5.96±0.15	7.10±0.15	ND	ND	NS		Amide	Amine-like
V7	3,5,5-Trimethyl-2-hexene	26456-76-8	ND	0.39±0.15	ND	ND	***		Alkene	Citrus, fruity
V31	3-Methyl-1,4-pentadiene	1115-08-8	ND	ND	ND	25.40±0.15		***	Alkene	Green
V34	3-Methylcyclopentene	1120-62-3	ND	ND	27.65±0.15	ND		***	Alkene	Green
V38	4,4-Dimethyl-1-pentene	762-62-9	0.22±0.15	ND	ND	ND	***		Alkene	Green
V43	3-Butenylbenzene	768-56-9	ND	0.67±0.15	ND	ND	***		Alkene	Floral, green
V44	4-Methyleneheptane	15918-08-8	ND	ND	5.60±0.15	ND		***	Alkene	Green
V62	(E)-2,2-Dimethyl-3-hexene	690-93-7	ND	ND	0.31±0.15	ND		***	Alkene	Green, fruity
V83	(Z)-4,4-Dimethyl-2-pentene	762-63-0	ND	ND	4.07±0.41	ND		***	Alkene	Green
V93	Acetohydroxamic acid	546-88-3	ND	ND	3.66±0.15	ND		***	Oxime	Aldehydic
V6	1-Isopropyl-1-methylcyclohexane	16580-26-0	0.25±0.15	0.31±0.15	ND	ND	NS		Alkane	Citrus
V41	4,5-Dimethyloctan	15869-96-2	ND	ND	ND	1.30±0.15		***	Alkane	Alkane-like

	e									
V56	Propylcyclopropane	2415-72-7	ND	1.76±0.29	10.30±2.66	6.27±0.15	***	*	Alkane	Alkane-like
V88	Neopentane	463-82-1	ND	ND	1.11±0.15	ND		***	Alkane	Alkane-like
V101	2-Cyclopropylbutane	1406223	0.26±0.15	ND	ND	0.28±0.15	***	***	Alkane	Alkane-like
V11	1,3-Bis(1,1-dimethylethyl)benzene	1014-60-4	ND	ND	0.58±0.15	0.34±0.15		*	Aromatic hydrocarbon	Woody
V1	3,5-Octadien-2-one	38284-27-4	ND	ND	0.81±0.32	ND		***	Ketone	Floral, fruity
V8	6-Oxa-bicyclo[3.1.0]hexan-3-one	74017-10-0	ND	3.47±0.15	ND	ND	***		Ketone	Ethereal
V30	(E,E)-3,5-Octadien-2-one	30086-02-3	ND	0.77±0.15	1.29±0.15	ND	***	***	Ketone	Floral, fruity
V35	3-Pentanone	96-22-0	ND	ND	0.31±0.15	0.73±0.15		**	Ketone	Ketonic, buttery
V36	3-Ethyl-2-cyclopenten-1-one	5682-69-9	ND	0.36±0.15	ND	ND	***		Ketone	Caramellic
V66	2,3,3-Trimethylcyclobutanone	28290-01-9	0.24±0.15	ND	ND	ND	***		Ketone	Ketonic, buttery
V87	3-Nonen-5-one	82456-34-6	ND	ND	0.45±0.15	ND		***	Ketone	Ketonic
V25	Methyltartronic acid	595-98-2	15.19±0.15	8.10±1.50	ND	ND	*		Carboxylic acid	Acidic
V54	Acetic acid	64-19-7	ND	15.67±0.15	7.64±0.15	7.85±1.87	***	NS	Carboxylic acid	Acetic
V58	Pyruvic acid	127-17-3	9.17±0.15	ND	ND	ND	***		Carboxylic acid	Acidic
V67	Hexanoic acid	142-62-1	ND	ND	0.51±0.27	0.43±0.15		NS	Carboxylic acid	Acidic, fatty
V99	Pentanoic acid	109-52-4	ND	0.89±0.15	0.67±0.15	ND	***	***	Carboxylic acid	Acidic, fatty
V59	Hydrogen azide	7782-79-8	4.63±0.15	ND	ND	ND	***		Acid	Pungent
V57	Propanoic anhydride	123-62-6	0.48±0.15	0.60±0.15	ND	0.39±0.15	NS	***	Anhydride	Acidic
V90	Acetic anhydride	108-24-7	ND	ND	ND	3.16±0.15		***	Anhydride	Anhydride
V20	2-Methyl-2H-tetrazole	16681-78-0	ND	ND	ND	5.85±0.15		***	Tetrazole	Herbal

V9	3-Ethyl-1H-1,2,4-triazole	7411-16-7	ND	0.37±0.15	ND	ND	***		Triazole	Herbal
V42	4H-1,2,4-Triazol-4-amine	584-13-4	ND	ND	2.06±0.27	ND		***	Triazole	Herbal
V78	1H-1,2,3-Triazole	288-36-8	ND	ND	ND	3.26±0.15		***	Triazole	Herbal
V76	Bis(1,1-dimethylethyl)diazene	927-83-3	ND	ND	ND	1.12±0.15		***	Azo compound	Chemical
V29	3,4,4-Trimethyl-2-azetidinone	22607-01-8	ND	0.25±0.15	ND	ND	***		Lactam	Dairy, nutty
V86	3,3-Diethyl-1-methyl-2,4-azetidinedione	69315-91-9	0.73±0.15	ND	ND	0.36±0.15	***	***	Lactam	Ketonic, buttery
V12	Cyano-3,4-epithiobutane	54096-45-6	0.31±0.15	0.39±0.15	ND	ND	NS		Nitrile	Sulfurous
V24	2-Methylhexanedinitrile	16525-39-6	ND	ND	33.24±12.69	ND		***	Nitrile	Chemical
V33	(CH3)2C=CHCN	4786-24-7	0.30±0.15	0.29±0.15	0.43±0.15	0.47±0.15	NS	NS	Nitrile	Green, fruity
V45	5-Methylhexanenitrile	19424-34-1	2.74±1.90	2.00±0.19	ND	ND	NS		Nitrile	Fruity, sweet
V46	5-(Methylthio)pentanenitrile	59121-25-4	25.11±12.65	20.14±3.19	0.48±0.15	0.56±0.29	NS	NS	Nitrile	Sulfurous, garlic
V52	Benzenepropanenitrile	645-59-0	6.08±2.69	5.58±0.76	0.85±0.36	0.33±0.15	NS	*	Nitrile	Almond-like
V77	Azeleonitrile	1675-69-0	ND	0.31±0.15	ND	ND	***		Nitrile	Nitrile, fatty
V94	Isoamyl cyanide	542-54-1	ND	0	2.50±0.15	ND		***	Nitrile	Nitrile-like
V95	Heptanonitrile	629-08-3	2.40±1.65	1.27±0.15	0.50±0.15	ND	*	***	Nitrile	Nitrile, fatty
V80	2,2',5,5'-Tetrahydro-2,2'-bifuran	98869-92-2	ND	ND	ND	1.83±0.15		***	Furan	Furan-like
V40	4,5-Dihydro-4,5-dimethyl-1H-pyrazole	28019-94-5	ND	2.57±0.15	ND	ND	***		Pyrazole	Herbal
V47	5-Ethyl-4,5-dihydro-3,5-dimethyl-1H-pyrazole	21981-22-6	ND	ND	0.51±0.15	ND		***	Pyrazole	Herbal
V1	2,6-	41536-80-	ND	0.34±0.	ND	ND	***		Amine	Amine-

8	Pyrazinediamine	5		15						like
V55	1-Methyl-2-methyleneaziridine	25012-55-9	ND	ND	8.42±0.15	ND		***	Amine	Amine-like
V49	Ammonium sulfamate	7773-06-0	0.99±0.15	ND	ND	ND	***		Ammonium salt	Amine-like
V50	Ammonium carbamate	1111-78-0	ND	0.35±0.15	ND	ND	***		Ammonium salt	Amine-like
V3	(E,E)-2,4-Heptadienal	881395	ND	0.48±0.15	0.75±0.15	ND	***	***	Aldehyde	Green, fatty
V19	2,6-Dimethyl-5-heptenal	106-72-9	ND	46.11±0.15	3.67±0.15	17.99±0.15	***	**	Aldehyde	Citrus, green
V21	2-Methyl-2-butenal	1115-11-3	ND	3.11±0.15	3.21±0.15	ND	***	***	Aldehyde	Green, fruity
V51	2-Butenal	4170-30-3	ND	1.77±0.15	2.09±0.29	ND	***	***	Aldehyde	Green, fruity
V53	Benzaldehyde	100-52-7	ND	0	0.70±0.15	ND		***	Aldehyde	Almond, sweet
V63	(E)-2-Hexenal	6728-26-3	2.10±0.15	0	0.40±0.15	ND	***	***	Aldehyde	Green, fruity
V97	Hexanal	66-25-1	ND	2.72±0.15	3.08±0.32	ND	***	***	Aldehyde	Green, fruity
V37	3-(1-Methylethyl)oxetane	10317-17-6	ND	ND	ND	0.43±0.15		***	Ether	Ethereal
V71	Dimethyl ether	115-10-6	18.32±9.32	12.64±1.89	8.70±1.83	8.04±0.15	*	NS	Ether	Ethereal
V16	Disulfide, methyl (methylthio)methyl	42474-44-2	0.35±0.15	0.41±0.16	ND	0.28±0.15	NS	***	Thioether	Garlic, sulfurous
V60	Disulfide, dimethyl	624-92-0	ND	ND	ND	22.01±0.15		***	Thioether	Sulfurous, garlic
V61	Dimethyl trisulfide	3658-80-8	8.64±0.15	12.98±1.64	6.30±0.15	4.60±0.25	*	*	Thioether	Sulfurous, garlic
V79	Monomethyl carbonotrithioate	1113-26-4	0.92±0.15	ND	ND	ND	***		Thiocarbonate	Sulfurous
V28	1-(2-Thienyl)-1-pentanone	53119-25-8	ND	ND	ND	1.66±0.15		***	Thioketone	Sulfurous, roasted meat
V13	Mercaptoacetone	24653-75-6	ND	10.41±0.15	ND	ND	***		Thiol	Sulfurous,

										roasted meat
V70	Methanethiol	74-93-1	2.89±0.15	4.09±0.15	3.79±0.15	4.08±2.02	*	NS	Thiol	Sulfurous, garlic
V14	1-Penten-3-ol	616-25-1	ND	1.26±0.15	1.40±0.22	ND	***	***	Alcohol	Green
V15	1-Octen-3-ol	3391-86-4	0.30±0.15	0.50±0.21	0.37±0.15	0.35±0.15	*	NS	Alcohol	Mushroom, earthy
V23	2-Methylcyclopentanol	24070-77-7	ND	0.70±0.15	ND	ND	***		Alcohol	Green, alcoholic
V27	2-Ethylcyclobutanol	35301-43-0	ND	ND	3.15±0.15	ND		***	Alcohol	Alcoholic
V32	3-Methyl-3-penten-1-ol	1708-99-2	ND	4.67±0.15	3.37±0.15	ND	***	***	Alcohol	Floral, fruity
V39	4,4-Dimethyl-2-pentanol	6144-93-0	ND	1.54±0.15	ND	ND	***		Alcohol	Alcoholic, fruity
V64	(E)-2-Hexen-1-ol	928-95-0	0.75±0.15	0.69±0.15	4.86±2.67	1.58±0.44	NS	*	Alcohol	Floral, green
V65	(E)-3-Hexen-1-ol	928-97-2	2.49±0.15	3.67±0.15	50.47±0.15	54.16±0.15	*	NS	Alcohol	Green, fruity
V69	4-Methyl-3-penten-1-ol	763-89-3	ND	ND	27.97±0.15	1.89±0.15		*	Alcohol	Floral, fruity
V81	(Z)-2-Penten-1-ol	1576-95-0	1.20±0.15	1.52±0.15	1.38±0.43	0.72±0.15	*	*	Alcohol	Floral, fruity
V82	(Z)-4-Hexen-1-ol	928-91-6	ND	ND	ND	30.41±0.15		***	Alcohol	Green, fruity
V84	(1R,2R)-2-Methylcyclopentanol	25144-05-2	ND	5.89±0.15	ND	ND	***		Alcohol	Alcoholic, fruity
V85	2-Amino-1,3-propanediol	534-03-2	4.42±0.15	ND	ND	ND	***		Alcohol	Sweet, amino acid-like
V96	1-Hexanol	111-27-3	1.76±0.15	5.78±5.23	ND	6.36±0.15	*	***	Alcohol	Alcoholic, sweet
V98	1-Pentanol	71-41-0	ND	ND	0.36±0.15	0.34±0.15		NS	Alcohol	Alcoholic, sweet

Note: Values are expressed as relative peak area (×10^6) and presented as mean ± standard deviation (SD) of three independent experiments (n=3). Statistical analysis: Independent samples t-test was performed to

compare non-fermented and L. rhamnosus-fermented samples within each tissue type (stalk or floret). Stalk Sig.: Significance between Control stalk and Lr fermented stalk; Floret Sig.: Significance between Control floret and Lr fermented floret. Significance levels: * $P < 0.05$, ** $P < 0.01$, *** $P < 0.001$ vs. corresponding non-fermented control; NS: Not Significant ($P \geq 0.05$). ND: not detected (below limit of detection, LOD). Aroma descriptions was obtained from published literature and Perflavory database (http://www.perflavory.com/, accessed on 24 April 2024).

Table S3. Screening of characteristic volatile components in broccoli florets and stalks (non-fermented).

No.	Compound	CAS	Category	Aroma	VIP	P-value	Log2FC	Significance
V1	3,5-Octadien-2-one	38284-27-4	Ketone	Floral, fruity	1.7175353	0.0459800	32.492106	*
V95	Heptanonitrile	629-08-3	Nitrile	Nitrile, fatty	1.7364532	0.0420008	-3.271879	*
V68	Methyl hexanoate	106-70-7	Ester	Fruity, sweet	1.7583456	0.0380105	33.082052	*
V81	(Z)-2-Penten-1-ol	1576-95-0	Alcohol	Floral, fruity	1.8207131	0.0270667	2.197892	*
V51	2-Butenal	4170-30-3	Aldehyde	Green, fruity	1.8343501	0.0263901	33.864890	*
V3	(E,E)-2,4-Heptadienal	881395	Aldehyde	Green, fatty	1.8394080	0.0257338	32.382494	*
V64	(E)-2-Hexen-1-ol	928-95-0	Alcohol	Floral, green	1.9444598	0.0133900	4.687077	*
V52	Benzenepropanenitrile	645-59-0	Nitrile	Almond-like	2.0140332	0.0084113	-2.839515	**
V46	5-(Methylthio)pentanenitrile	59121-25-4	Nitrile	Sulfurous, garlic	2.0263741	0.0075308	-7.705143	**
V75	Methyl thiocyanate	556-64-9	Ester	Sulfurous	2.1827139	0.0012688	32.819766	**

Note: Characteristic volatile components were screened based on VIP > 1 and $P < 0.05$ using OPLS-DA analysis. VIP: Variable Importance in Projection; Log2FC: Log2 fold change (floret/stalk). Positive Log2FC indicates higher content in florets; negative Log2FC indicates higher content in stalks. Statistical significance was determined by independent samples t-test (n=3). Significance levels: * $P < 0.05$, ** $P < 0.01$, *** $P < 0.001$.

Table S4. Screening of characteristic volatile components in non-fermented and Lr fermented broccoli stalks.

No.	Compound	CAS	Category	Aroma	VIP	P-value	Log2FC	Significance
V36	3-Ethyl-2-cyclopenten-1-one	5682-69-9	Ketone	Caramellic	1.2507282	0.0019000	29.734194	**
V9	3-Ethyl-1H-1,2,4-triazole	7411-16-7	Triazole	Herbal	1.3615173	0.0023000	29.799175	**
V77	Azeleonitrile	1675-	Nitrile	Nitrile,	1.2201753	0.0023000	29.506298	**

		69-0		fatty				
V50	Ammonium carbamate	1111-78-0	Ammonium salt	Amine-like	1.2201753	0.0033000	29.717846	**
V63	(E)-2-Hexenal	6728-26-3	Aldehyde	Green, fruity	1.2161338	0.0035000	- 32.289789	**
V7	3,5,5-Trimethyl-2-hexene	26456-76-8	Alkene	Citrus, fruity	1.8346990	0.0043000	30.862579	**
V22	2-Methylbutyl acetate	624-41-9	Ester	Fruity, sweet	1.2161338	0.0050000	- 33.915371	**
V79	Monomethyl carbonotrithioate	1113-26-4	Thiocarbonate	Sulfurous	1.2166777	0.0109000	- 31.093819	*
V21	2-Methyl-2-butenal	1115-11-3	Aldehyde	Green, fruity	1.3440344	0.0171000	32.856832	*
V30	(E,E)-3,5-Octadien-2-one	30086-02-3	Ketone	Floral, fruity	1.3440344	0.0192000	30.847153	*
V29	3,4,4-Trimethyl-2-azetidinone	22607-01-8	Lactam	Dairy, nutty	1.2201753	0.0277000	29.244413	*
V75	Methyl thiocyanate	556-64-9	Ester	Sulfurous	1.3440344	0.0285000	30.841397	*
V96	1-Hexanol	111-27-3	Alcohol	Alcoholic, sweet	1.4257205	0.0293000	2.715176	*
V86	3,3-Diethyl-1-methyl-2,4-azetidinedione	69315-91-9	Lactam	Ketonic, buttery	1.2166777	0.0298000	- 30.766050	*
V14	1-Penten-3-ol	616-25-1	Alcohol	Green	1.3440344	0.0395000	31.527765	*
V101	2-Cyclopropylbutane	1406223	Alkane	Alkane-like	1.2484637	0.0412000	- 29.284620	*
V49	Ammonium sulfamate	7773-06-0	Ammonium salt	Amine-like	1.2166777	0.0431000	- 31.199942	*
V32	3-Methyl-3-penten-1-ol	1708-99-2	Alcohol	Floral, fruity	1.2507282	0.0449000	33.443135	*
V2	Methyl 3-methylpentanoate	2177-78-8	Ester	Fruity, sweet	1.3440344	0.0476000	31.619375	*

Note: Characteristic volatile components were screened based on VIP > 1 and *P* < 0.05 using OPLS-DA analysis. VIP: Variable Importance in Projection; Log2FC: Log2 fold change (Lr fermented stalk/non-fermented stalk). Positive Log2FC indicates up-regulation after fermentation; negative Log2FC indicates down-regulation after fermentation. Statistical significance was determined by independent samples t-test (n=3). Significance levels: * *P* < 0.05, ** *P* < 0.01, *** *P* < 0.001.

Table S5. Screening of characteristic volatile components in non-fermented and Lr fermented broccoli florets.

No.	Compound	CAS	Category	Aroma	VIP	P-value	Log2FC	Significance
V70	Methanethiol	74-93-1	Thiol	Sulfurous,	1.5386179	0.0188537	2.107528	*

				garlic				
V89	4-Hexen-1-yl acetate	72237-36-6	Ester	Fruity, floral	1.7055988	0.0203652	2.086361	*
V54	Acetic acid	64-19-7	Carboxylic acid	Acetic	1.6537353	0.0285922	2.039674	*
V81	(Z)-2-Penten-1-ol	1576-95-0	Alcohol	Floral, fruity	1.8651266	0.0172846	-2.528847	*
V75	Methyl thiocyanate	556-64-9	Ester	Sulfurous	1.8738460	0.0162688	-2.789924	*
V95	Heptanonitrile	629-08-3	Nitrile	Nitrile, fatty	1.2732269	0.0440862	-31.210440	*
V99	Pentanoic acid	109-52-4	Carboxylic acid	Acidic, fatty	1.2513456	0.0458652	-31.638200	*
V92	(Z)-3-Hexen-1-yl acetate	3681-71-8	Ester	Green, fruity	1.2451258	0.0439259	-31.875200	*
V3	(E,E)-2,4-Heptadienal	881395	Aldehyde	Green, fatty	1.6818655	0.0251516	-32.382490	*
V1	3,5-Octadien-2-one	38284-27-4	Ketone	Floral, fruity	1.5518445	0.0166883	-32.492110	*
V30	(E,E)-3,5-Octadien-2-one	30086-02-3	Ketone	Floral, fruity	1.2514860	0.0457159	-32.585250	*
V14	1-Penten-3-ol	616-25-1	Alcohol	Green	1.2489203	0.0480111	-32.702090	*
V68	Hexanoic acid, methyl ester	106-70-7	Ester	Fruity, sweet	1.6158655	0.0052184	-33.082050	**
V42	4H-1,2,4-Triazol-4-amine	584-13-4	Triazole	Herbal	1.2712606	0.0462933	-33.260650	*
V97	Hexanal	66-25-1	Aldehyde	Green, fruity	1.2724567	0.0451899	-33.841700	*
V51	2-Butenal	4170-30-3	Aldehyde	Green, fruity	1.6753208	0.0262700	-33.864890	*
V83	(Z)-4,4-Dimethyl-2-pentene	762-63-0	Alkene	Green	1.2090779	0.0450621	-34.242640	*

Note: Characteristic volatile components were screened based on VIP > 1 and $P < 0.05$ using OPLS-DA analysis. VIP: Variable Importance in Projection; Log2FC: Log2 fold change (Lr fermented floret/non-fermented floret). Positive Log2FC indicates up-regulation after fermentation; negative Log2FC indicates down-regulation after fermentation. Statistical significance was determined by independent samples t-test (n=3). Significance levels: * $P < 0.05$, ** $P < 0.01$, *** $P < 0.001$.

Table S6. Screening of characteristic volatile components in fermented broccoli florets and stalks.

No.	Compound	CAS	Category	Aroma	VIP	P-value	Log2FC	Significance

V25	Methyltartronic acid	595-98-2	Carboxylic acid	Acidic	1.3870488	0.0095985	-35.236720	**
V45	5-Methylhexanenitrile	19424-34-1	Nitrile	Fruity, sweet	1.9052169	0.0340666	-33.804050	*
V95	Heptanonitrile	629-08-3	Nitrile	Nitrile, fatty	1.9029681	0.0344723	-33.146140	*
V12	Cyano-3,4-epithiobutane	54096-45-6	Nitrile	Sulfurous	1.8801070	0.0391813	-31.434740	*
V6	1-Isopropyl-1-methylcyclohexane	16580-26-0	Alkane	Citrus	1.3927090	0.0080310	-30.536330	**
V46	5-(Methylthio)pentanenitrile	59121-25-4	Nitrile	Sulfurous, garlic	1.8664241	0.0421505	-5.346268	*
V52	Benzenepropanenitrile	645-59-0	Nitrile	Almond-like	1.8471457	0.0462290	-4.260626	*
V81	(Z)-2-Penten-1-ol	1576-95-0	Alcohol	Floral, fruity	1.6124473	0.0283333	-2.257236	*
V64	(E)-2-Hexen-1-ol	928-95-0	Alcohol	Floral, green	2.2117847	0.0118314	2.197223	*
V89	4-Hexen-1-yl acetate	72237-36-6	Ester	Fruity, floral	2.4835755	0.0002262	34.742653	***

Note: Characteristic volatile components were screened based on VIP > 1 and $P < 0.05$ using OPLS-DA analysis. VIP: Variable Importance in Projection; Log2FC: Log2 fold change (fermented floret/fermented stalk). Positive Log2FC indicates higher content in fermented florets; negative Log2FC indicates higher content in fermented stalks. Statistical significance was determined by independent samples t-test (n=3). Significance levels: * $P < 0.05$, ** $P < 0.01$, *** $P < 0.001$.

Table S7. Sensory evaluation scores of broccoli juices before and after fermentation.

Sensory attribute	Stalk		Floret	
	Non-fermented	Fermented	Control	Non-fermented
Color	5.11±1.36	4.67±1.50^{ns}	6.11±0.93	5.56±0.73^{ns}
Texture	6.11±2.03	6.33±1.66^{ns}	5.67±0.50	6.22±1.39^{ns}
Aroma	4.78±2.11	4.11±0.93^{ns}	5.67±1.50	5.89±0.60^{ns}
Oral texture	5.89±1.96	4.22±1.48^{ns}	6.00±1.22	5.89±0.60^{ns}
Overall acceptance	6.56±1.88	5.89±0.78^{ns}	6.00±0.87	6.00±1.12^{ns}

Table note: Data are presented as mean ± standard deviation. ns indicates no significant difference between the treatment group and its corresponding control group (independent sample t-test, P>0.05, n=9).
